# Targeting the vector of arboviruses *Aedes aegypti* with nanoemulsions based on essential oils: a review with focus on larvicidal and repellent properties

**DOI:** 10.3762/bjnano.16.132

**Published:** 2025-10-28

**Authors:** Laryssa Ferreira do Nascimento Silva, Douglas Dourado, Thayse Silva Medeiros, Mariana Alice Gonzaga Gabú, Maria Cecilia Queiroga dos Santos, Daiane Rodrigues dos Santos, Mylena Lemos dos Santos, Gabriel Bezerra Faierstein, Rosângela Maria Rodrigues Barbosa, Fabio Rocha Formiga

**Affiliations:** 1 Department of Immunology, Aggeu Magalhães Institute (IAM), Oswaldo Cruz Foundation (FIOCRUZ), 50670-420 Recife, PE, Brazil; 2 Department of Entomology, Aggeu Magalhães Institute (IAM), Oswaldo Cruz Foundation (FIOCRUZ), 50670-420 Recife, PE, Brazil; 3 Faculty of Medical Sciences (FCM), University of Pernambuco (UPE), 50100-130, Recife, PE, Brazil

**Keywords:** *Aedes aegypti*, arboviruses, mosquito vector, nanoemulsion, nanotechnology, natural products

## Abstract

Mosquitoes of the *Aedes* genus are responsible for the transmission of arboviruses that seriously affect public health. Given the increasing resistance to traditional insecticides and their negative environmental impacts, the need for safer alternatives arises. In this context, natural produts such as essential oils (EOs) have been studied for their larvicidal and repellent properties against *Aedes aegypti*, due to the presence of compounds such as terpenoids and phenols. However, the usage of EOs is limited due to some properties such as poor water solubility, high volatility, and intrinsic oxidation sensitivity. Thus, the development of novel formulations to efficiently deliver bioactives represents an innovative approach for *Aedes aegypti* control. In this context, nanothecnology provides smart formulations with improved drug solubility, controlled release, and protection against degradation. Nanoemulsions are colloidal systems with droplets of 20 to 500 nm, which improve the dispersion of the compounds, protect their active properties, and enhance their efficacy. This review addresses the potential of nanoemulsions as efficient carriers of EOs, and how this approach could emerge as ecological alternatives to synthetic insecticides. Herein, the focus was kept on targeting larvicidal and repellent activities against *Ae. aegypti*. For that, 23 studies were analyzed, which demonstrated a significant increase in the efficacy of nanoemulsions with EOs compared to that of free EOs, in both activities. However, the repellent activity has been less explored, present in only three of the studies evaluated, in the last 10 years. Correlatingh with this, other aspects such as botanical species of EOs, mechanisms of action, composition, and characteristics of nanoemulsions are discussed. In addition, this review highlights challenges and perspectives on pharmaceutical nanotechnology towards nanoemulsions as safe, effective, and eco-friendly tools for controlling *Ae. Aegypti.*

## Introduction

Arboviruses (arthropod-borne viruses) are viruses transmitted by arthropods, including mosquitoes, sandflies, and ticks. Currently, more than 615 arboviruses have been recognized and reported in the literature. Their transmission cycle typically begins when a vector, such as a mosquito, feeds on the blood of an infected host [1]. Thus, the virus undergoes a replication process in the midgut of the mosquito, being disseminated to different organs, mainly the salivary glands. Upon contact with a new host, the virus is inoculated through the bite of the vector, continuing the transmission cycle [2–3]. Most diseases caused by arboviruses are zoonoses, meaning they are primarily infections of vertebrates which can occasionally trigger incidental infections in humans [3–4].

The main vectors are mosquitoes of the *Aedes* genus, primarily *Aedes aegypti* and *Aedes albopictus*, which are capable of transmitting various arboviruses on a global scale [5]. *Ae. aegypti* is generally considered the most efficient and important vector for the transmission of these viral diseases, due to its evolutionary adaptations, its strong association with human environments, and its high vector competence [5–6]. However, *Ae. albopictu*s has been reported as the main or even the sole vector responsible for arbovirus transmission in certain regions of the world, demonstrating significant epidemiological relevance under specific ecological conditions [7]. Therefore, these arthropods play a crucial role in the transmission of arboviruses of global epidemiological relevance, such as dengue (DENV), yellow fever (YFV), zika (ZIKV), and chikungunya (CHIKV).

In these diseases, high morbidity and mortality rates are observed, and they have a global distribution, particularly in tropical and subtropical regions, where climatic conditions favor the reproduction and proliferation of mosquitoes. Due to the growing threat posed by arboviruses, the Global Arbovirus Initiative [8] highlights integrated vector control as a critical pillar for reducing transmission risks worldwide, advocating for the use of multiple complementary approaches as an essential strategy for effective disease control. These include the rational use of chemical insecticides, aimed at minimizing the development of resistance; biological control, through the use of natural predators and larvicidal microorganisms such as *Bacillus thuringiensis israelensis* (Bti); mechanical control, based on improving basic sanitation and infrastructure to eliminate breeding sites; and behavioral control, which promotes the adoption of practices such as the use of traps to capture mosquitoes at different life stages [9–12]. In this context, the development of nanoemulsions based on essential oils emerges as a promising innovation within integrated control strategies, offering environmentally friendly larvicidal and repellent alternatives specifically targeted at *Aedes aegypti* [12–14].

Although chemical insecticides are important tools in vector control, the development of resistance and the consequent decrease in their effectiveness have become a major concern [15–18]. Several cases of resistance to pyrethroids, carbamates, organochlorines, and organophosphates have been reported over the years [11,19]. Additionally, repellents based on DEET (*N*,*N*-diethyl-meta-toluamide) are recognized as the gold standard for mosquito repellents; however, these repellents are not widely used in regions at risk for arbovirus transmission, mainly due to accessibility issues, cost, and concerns about long-term safety. Additionally, commonly used components have been associated with allergic reactions, skin irritation, and adverse effects on the nervous, cardiovascular, and respiratory systems of humans because these chemicals are highly toxic to non-target organisms and are not selective for the vector [11,20–21]. Furthermore, these products also contribute to environmental pollution [14]. Given this scenario, the search for more sustainable alternatives, such as the use of natural products, becomes a promising strategy to tackle these challenges [22].

Among natural products are essential oils, secondary metabolites with complex chemical composition extracted from different parts of plants [23]. They stand out for their efficiency in combating the vector at different stages of the evolutionary cycle, especially as larvicides, wherein the mosquito is at its most vulnerable stage [24]. In addition, they can act as insecticides, ovicides, pupicides, oviposition deterrents, and repellents [25–26]. The main advantages include low toxicity, biodegradability, and action in multiple locations due to the variety of compounds [12,19].

On the other hand, essential oils in their natural form are not stable under environmental stress, being easily degradable or evaporated upon exposure to air, light, heat, and humidity during processing, use, or storage [27–28]. Their volatile nature, susceptibility to oxidation, and insolubility in water limit their industrial application [29]. To overcome these limitations, nanotechnological strategies have been used, such as polymeric nanocarriers [30], solid lipid nanoparticles [31], liposomes [32], and nanoemulsions [13–14,33].

Among these strategies, nanoemulsions, kinetically stable nanometric dispersions (20–500 nm) of two immiscible liquids, stabilized by surfactants, have stood out [34]. These are not affected by moderate changes in pH or temperature, making them ideal for the protection of solubilized bioactives [35]. They also promote the protection of essential oils against oxidation caused by external factors, maintaining or increasing their functional properties [28]. Furthermore, they increase the water solubility of poorly soluble compounds, improve the dispersion of essential oils in vector control, and provide a controlled release of the bioactives. Finally, these systems can be obtained at low cost and through more sustainable technologies [36].

Given these advantages, nanoemulsions containing essential oils have been used as a promising strategy for mosquito control [33]. These systems can improve the efficacy of essential oils (EOs) against these vectors, providing the abundance of these bioactives in their structures at different stages of their cycle [37]. Therefore, this review focuses on mapping nanoemulsions based on essential oils and their potential as an innovative strategy for controlling *Aedes aegypti* and consequently related arboviruses.

## Review

### *Aedes aegypti*: General aspects and control strategies

Insects are important vectors in the transmission of bacteria and viruses, contributing to the spread of various diseases in humans. Among these pathogens, arboviruses form a diverse group of viruses primarily transmitted by arthropods such as mosquitoes, ticks, and flies [38]. More than 500 arboviruses have been identified globally, but only about 150 cause diseases in humans. Among them, the *Flaviviridae* family includes pathogens such as dengue (DENV) and zika (ZIKV) viruses, while *Alphaviruses* include the chikungunya virus (CHIKV) [39–40]. These viruses are primarily transmitted by the *Ae. aegypti* mosquito, which relies on humans as its main amplification hosts [2,41–42].

*Aedes aegypti* is a species of mosquito of the *Culicidae* family, order *Diptera*, native to Africa and distributed throughout various regions of the world. This extensive distribution occurs due to the sum of several factors, such as dispersion favored by high temperatures and high humidity, and inefficient urban and rural development planning measures. Thus, due to climatic and social conditions, tropical and subtropical regions are considered favorable for the development of this mosquito [43].

**Figure 1:**
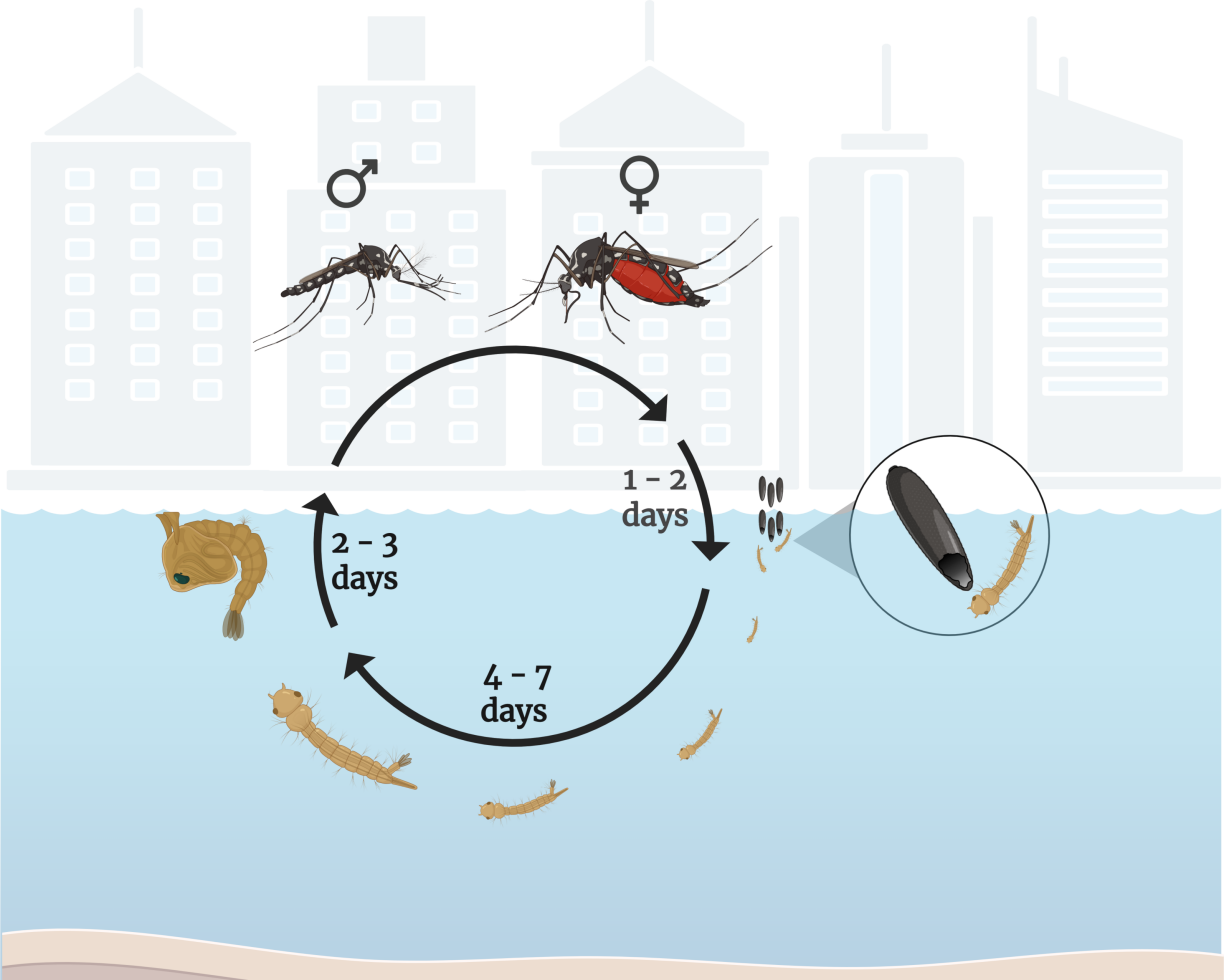
Life cycle of *Aedes aegypti* mosquitoes. After oviposition, the eggs hatch into larvae within one to two days. Larvae develop through four instar stages over four to seven days before becoming pupae. Pupae mature into adult mosquitoes within two to three days. Males usually emerge first. Only females need to feed on blood to develop their eggs and continue the cycle. The scale of the image in cm provides a reference for the approximate size of the mosquitoes and their stages of development. Created in BioRender. Rocha Formiga, F. (2025) https://BioRender.com/ssghykj. This content is not subject to CC BY 4.0.

*Ae. aegypti* mosquitoes are small in size, black in color, and have white spots [44]. Regarding their life cycle, they present complete metamorphosis, including the egg, larva, pupa, and adult stages (Figure 1) [45]. The complete cycle, which takes approximately seven to 14 days, encompasses the transition from an *Ae. aegypti* mosquito egg to an adult mosquito, and it is influenced by environmental factors such as temperature, nutrient availability, water quality, and ecological interactions. Higher temperatures accelerate growth, while food scarcity and competition can prolong this phase [46–48].

The life cycle of *Ae. aegypti* (Figure 1) begins with the deposition of eggs by adult female mosquitoes. These eggs are capable of surviving for a prolonged period in the absence of water and can withstand extreme environmental conditions, thereby persisting in aridand/or cold environments [20,48]. Upon contact with water, the eggs hatch, resulting in the hatching of larvae corresponding to the 1st instar stage (L1). Larvae rapidly progress to the 2nd instar stage (L2), and subsequently to the 3rd instar stage (L3) larvae. The transition from L3 to the 4th instar stage (L4) occurs more gradually, as larvae must achieve sufficient size and weight to initiate metamorphosis into pupae. Pupae also live exclusively in aquatic habitats and represent a transitional phase of metamorphosis between larval and adult stages. During this phase, pupae develop into adult mosquitoes, which are capable of flying short distances. *Aedes aegypti* mosquitoes generally disperse within 50 to 100 meters from their emergence site [49], although in less populated areas this range may extend up to 560 meters in rural or more open environments [45,48,50–51].

Environmental factors, such as improper water storage and climatic conditions, influence the proliferation of *Ae. aegypti,* increasing arbovirus transmission. According to data from the World Health Organization [52], arboviroses are seen as a major obstacle to public health, especially to dengue, zika, and chikungunya viruses, especially in subtropical and tropical countries [3]. In this scenarium, DENV causes more than 400 million infections annually, presenting symptoms such as high fever, headaches, and, in severe cases, hemorrhagic shock [53]. CHIKV, present in over 100 countries, leads to fever, severe joint pain, and, in critical cases [54–55], to neurological and cardiac complications [55]. Initially overlooked, ZIKV gained global attention after outbreaks in the Americas, being linked to severe complications such as congenital zika syndrome and Guillain–Barré syndrome [56]. In addition to mosquito transmission, ZIKV can spread through sexual contact [57], blood transfusions [58], and from mother to child [59].

Given the persistence and severity of arboviral diseases, effective strategies to control *Ae. aegypti* are crucial for preventing their spread. The absence of vaccines and effective treatments for most arboviruses further reinforces the importance of vector control as an important strategy to reduce the transmission of these diseases [60]. For that, a variety of methods targeting different stages of the mosquito life cycle have been employed to control their populations. Traditional strategies focus on eliminating breeding sites, such as water containers and discarded tires that collect rainwater, which serve as ideal habitats for mosquito larvae. Additionally, the application of larvicides, including chemical and biological agents, insect growth regulators, and bacterial toxins, has been widely used to reduce mosquito populations. Internal residual spraying to eliminate adult mosquitoes and the use of insecticide-treated nets are also common control measures [61–62].

Historically, the Pan American Health Organization (PAHO), in collaboration with Latin American governments, led efforts to eradicate *Ae. aegypti* in the Americas during the 1950s and 1960s, primarily using dichloro-diphenyl-trichloroethane (DDT) and other persistent insecticides. Although effective at the time, DDT use was discontinued due to its environmental persistence and the emergence of resistance in mosquito populations [60,63]. Another widely used insecticide is temephos, an organophosphate larvicide applied in water reservoirs to control mosquito reproduction. However, its direct correlation with reduced dengue cases remains inconclusive [60].

Despite their effectiveness, the excessive use of chemical insecticides poses health risks and contributes to the development of resistance in mosquito populations [63]. These concerns highlight the need for safer, more sustainable vector control strategies that minimize risks to human health and delay the onset of insecticide resistance. In this context, natural products have emerged as a promising alternative for mosquito control, particularly as larvicides, given that many insecticides are derived from natural compounds [64]. Various classes of natural products and secondary metabolites have been evaluated, with a particular focus on plant essential oils and their constituents [65–66].

### Essential oils in vector control

Essential oils are hydrophobic, aromatic, volatile liquid compounds mainly secreted by glandular trichomes, which are the secretory tissues present in different vegetative parts of plants, such as leaves, flowers, roots, fruits, or stems [67]. These can be obtained by distillation with water or steam, microwave or ultrasound assisted extraction, or from the epicarp of fruits by a mechanical process, or by “dry distillation”. They and are made of a complex mixture of low molecular weight chemical substances including terpenes, terpenoids, phenylpropanoids, aldehydes, ketones, and ethers which are directly related to the defense of plants against different pathogens [68–70].

The chemical compounds present in the essential oils of some plant species (such as *Cymbopogon excavatus*, *Mentha piperita*, *Azadirachta indica*, *Eucalyptus maculata*, and *Cymbopogon nardus*) [62,66], exhibit, either alone or in synergy, larvicidal, ovicidal, pupicidal, and repellent properties. These characteristics make these oils sustainable and effective alternatives for controlling disease-carrying insects, including *Ae. aegypti* [71–72]. Chemical components such as limonene, menthol, linalool, thymol, eugenol, citral, and camphor are widely used in commercial insecticide and repellent formulations around the world [67,73–74]. These constituents and others have been found in the composition of different species producing EOs, presenting significant biological activities against *Ae. aegypti* [72,75–77].

The action of these components consists of interfering with larval physiology, preventing its development and contributing to the reduction of the adult mosquito population. Ovicidal activities are also reported, which prevent eggs from hatching, interrupting the life cycle of the vector. Furthermore, essential oils exhibit repellent properties that keep mosquitoes away, reducing the incidence of bites and, consequently, the transmission of diseases [10,24].

Despite the potential of essential oils, their use in vector control faces several limitations. The high volatility of the active compounds can reduce long-term efficacy, requiring frequent reapplications. In addition, the low polarity of the components of essential oils makes their use unfeasible in aqueous environments, which are common for larval development. Furthermore, chemical instability and variation in the consistency of the composition of the oils can affect the efficacy of the products [78].

To overcome these limitations, pharmaceutical nanotechnology strategies such as nanoemulsions have been used as tools for vector control [79]. Numerous studies have shown that nanoemulsions can increase the solubility and prolong the larvicidal and/or repellent activity of essential oils, making them a promising solution to improve the efficacy of herbal products for mosquito control [80–81].

### Nanoemulsions: Concepts and applications in larvicides and repellents

Pharmaceutical nanotechnology offers innovative solutions for the delivery and targeting of molecules for therapeutic, prophylactic, or diagnostic purposes [82]. In 1995, the Food and Drug Administration (FDA) agency approved the first nanomedicine, Doxil^®^ (doxorubicin-loaded liposomes) for chemotherapy. Over the past 30 years, research and development in nanotechnology have expanded significantly, with more than 70 nanomedicines approved by FDA or EMA [83–86].

**Figure 2:**
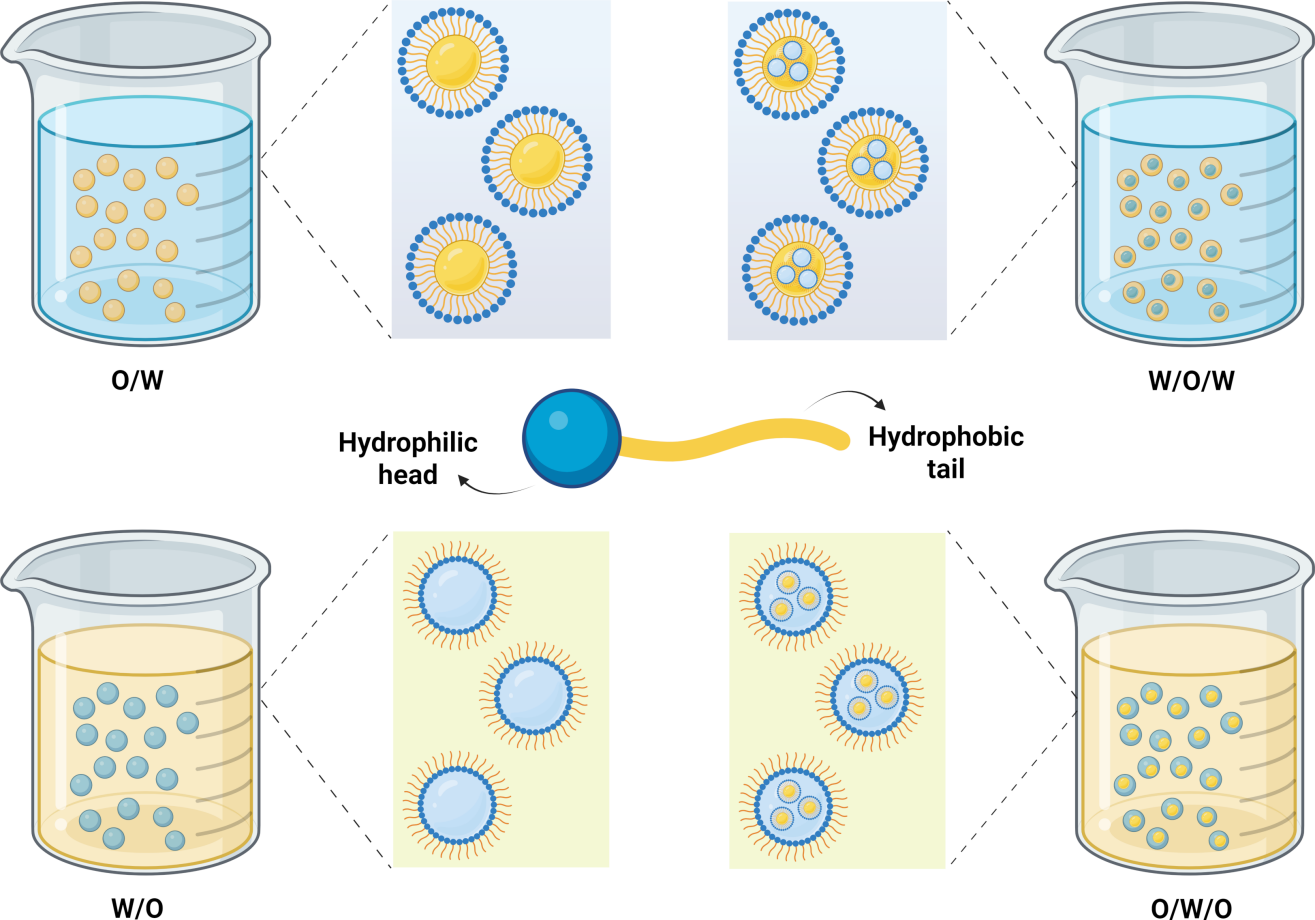
Nanoemulsion classification. Created in BioRender. Rocha Formiga, F. (2025) https://BioRender.com/mcdrou6. This content is not subject to CC BY 4.0.

Beyond liposomes, other lipid-based nanosystems have gained prominence, such as nanoemulsions (NEs) [87]. NEs are kinetically stable dispersed systems composed of two immiscible phases, typically an oil and aqueous phases, with droplets (20–500 nm) stabilized by surfactants. NEs can be classified as oil-in-water (O/W), water-in-oil (W/O), or multiple emulsions (W/O/W or O/W/O) (Figure 2), depending on the preparation techniques and the choice and interaction of formulation components [34,88–89].

Nanoemulsions exhibit several advantages, making them promising systems for pharmaceutical and biomedical applications. These include: (a) superior stability during storage compared to that of macroemulsions, attributed to their small droplet size, which prevents flocculation, creaming, and sedimentation; (b) enhanced bioavailability and improved aqueous solubility of lipophilic molecules; (c) increased cutaneous permeability of various molecules; (d) reduced emulsifier concentrations compared to that of macro- and microemulsions; (e) protection of molecules from adverse environmental conditions such as pH-induced hydrolysis and oxidation; and (f) versatile applications allowing administration in various forms, including gels, creams, aerosols, and sprays, via oral, topical, intravenous, pulmonary, and ocular routes [88,90–93].

Given these numerous advantages, nanoemulsions have been successfully explored for the treatment of diverse cancers [94], inflammatory processes [95], photodynamic therapy [96], antimicrobial applications [97], intracellular parasites such as *Trypanosoma cruzi* and *Leishmania spp*. [98–99], and for antioxidant potential [100], larvicidal, and repellent activities against arthropod vectors [101]. Nanotechnology has shown the potential to enhance the performance and significantly improve the physical, chemical, and biological properties of essential oils, phytocompounds, and other insecticidal materials [102].

Wei et al. (2023) demonstrated the larvicidal potential of fenpropathrin NEs against *Helicoverpa armigera* larvae at the L3 stage, reporting superior efficacy compared to commercial emulsions, along with lower toxicity to non-target organisms and improved environmental compatibility [103].

Similarly, Shaari et al. (2021) developed palm-oil-based nanoemulsions containing deltamethrin, which exhibited enhanced insecticidal performance against *Ae. aegypti* mosquitoes using thermal fogging techniques in outdoor environments [104]. This superior performance was attributed to the reduced droplet size, which facilitates penetration through mesothoracic spiracles and into the inner walls of the mesothoracic trachea, thereby increasing insect mortality.

Against the *Ae. aegypti* vector, Duarte et al. (2024) developed NEs loaded with two monoterpenes, cymene and myrcene, and evaluated their larvicidal potential against L3 larvae [36]. The NEs showed significant lethality to *Ae. aegypti* larvae, with insecticidal activity equal to or greater than that of free terpenes but with enhanced safety for *Galleria mellonella* larvae and human keratinocyte cells (HaCaT). Additionally, NEs facilitated dispersion in aqueous environments.

In conclusion, nanoemulsions are safe, eco-friendly, and effective nanosystems capable of enhancing the larvicidal and insecticidal effects of various compounds. Developing essential oil-based nanoemulsions represents an intelligent and promising alternative for vector control, as further detailed in the following sections.

### Nanoemulsions based on essential oils to control *Aedes aegypti*

There have been reports in recent years regarding the use of nanoemulsions as effective active EO carriers. In this review, we compiled studies of nanoemulsions containing essential oils against *Ae. aegypt*i, a vector of arboviruses such as zika, chikungunya, and dengue. We found 23 studies with different types of nanoemulsified essential oils. Most of the studies were related to larvicidal activity, with less investigation regarding repellent potential in recent years. These aspects are detailed and discussed in the sections below.

#### Larvicidal properties

**Figure 3:**
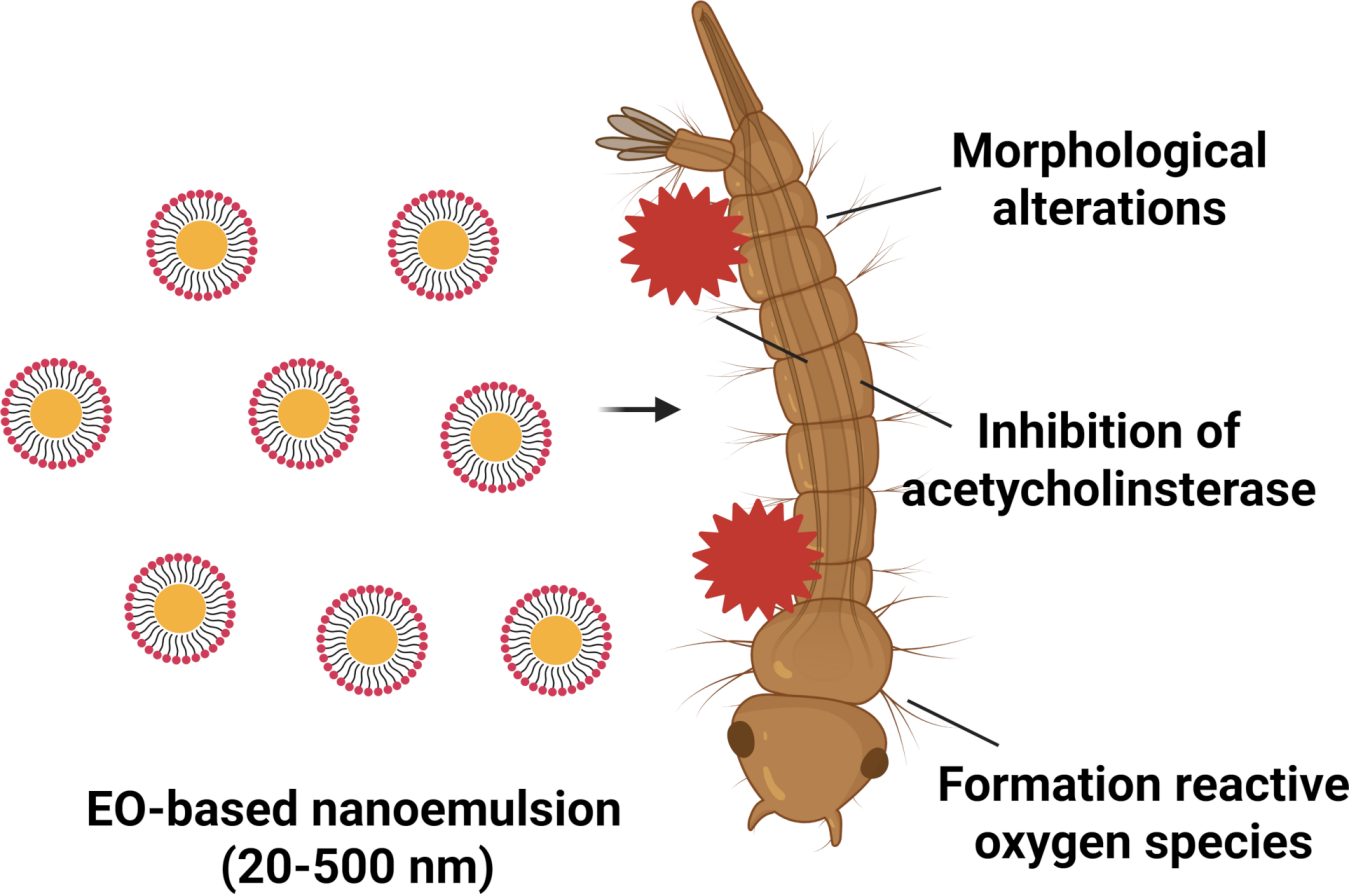
Schematic representation of the larvicidal mechanism of nanoemulsions based on essential oils against *Aedes aegypti* larvae. Created in BioRender. Rocha Formiga, F. (2025) https://BioRender.com/qu9l3j9. This content is not subject to CC BY 4.0.

Essential oils have gained attention due to their larvicidal properties against different vector larvae, including *Ae. aegypti*, and have been enhanced in nanoemulsions thanks to their properties [105–107]. Nanostructured plant-based larvicides could be associated with larvae morphological alterations, formation of reactive oxygen species that cause genotoxicity, and inhibition of acetylcholinesterase (Figure 3).

In this context, Romano et al. (2024) investigated the use of a nanoemulsion formulated with an essential oil extracted from the leaves of *Murraya koenigii* to evaluate its efficacy as a larvicide against the *Ae. aegypti* mosquito [108]. This plant, known as curry in India, is widely distributed in tropical and subtropical regions of the world and has culinary and medicinal applications [109]. Its leaves can be used to extract essential oils rich in sesquiterpenes, whose major components are β-phellandrene (38.3%), α-fenchene (10.5%), and sabinene (9.6%), which are important for its larvicidal activity [108]. Thus, a nanoemulsion with an average droplet size of 140 nm containing 1% of essential oils was evaluated on third-instar larvae (treated for 24 hours) at 1:100 and 1:200 (v:v) dilutions. The results reveal high mortality at different concentrations, with LC_50_ values of 11.8 µg/mL and 12.5 µg/mL, LC_90_ values of 22.6 µg/mL and 21.6 µg/mL for laboratory and field, respectively. In comparison to the unformulated essential oil, the nanoemulsion maintained its effectiveness for an extended period of up to 29 days. While genotoxic effects were not observed in *Allium cepa* cells, a decrease in cellular proliferation was evidenced.

De Sousa dos Santos et al. (2024) investigated the larvicidal effects of a nanoemulsion containing essential oil from *Ocimum basilicum* [76]. This species is known as basil or alfavaca and it is native to tropical regions of Africa and Asia [110]. It presents high levels of linalool (32.66%) and anethole (32.48%), in addition to α-selinene (0.82%) in their composition, which showed promising molecular affinity in in silico studies, despite its low concentration [76]. The oil was obtained by hydrodistillation and incorporated into the nanoemulsion through a low-energy method, using polysorbate 20 as a surfactant. The formulation presented an average droplet size ranging from 244.6 to 280.4 nm, a polydispersity index of less than 0.25, and a negative zeta potential (−15.7 to −18.6 mV), maintaining stability for 14 days. In the bioassays, fourth-stage larvae (L4) of *Aedes aegypti* and *Culex quinquefasciatus* were exposed to concentrations of 10 to 50 mg/L for 24 and 48 hours. LC_50_ values ranged from 38 to 42 mg/L for both vectors throughout the evaluated periods. Distilled water, polysorbate 20, and commercial larvicides (Temephos and Pyriproxyfen) were used as controls. It should be noted that the study did not perform comparisons with the free essential oil nor did it evaluate the formulation in non-target species [111–112].

Machado et al. (2023) developed an oil-in-water nanoemulsion containing 5% of surfactants and 5% of the essential oil of *Ocotea indecora* leaves and evaluated the larvicidal activity against *Ae. Aegypti* [113]. This species is an endemic plant native to Brazil found in the remaining Atlantic Forest in the southern and southeastern regions of Brazil [113–114]. Among these metabolites, there are reports of bicyclogermacrene, valerianol, β-pinene, and sesquirosefuran being the major substances in the essential oil of leaves (81.4%), suggesting a key role of this metabolites in larvicidal activity [113,115]. The oil was extracted using hydrodistillation and incorporated into the nanoemulsion by the low-energy method by phase inversion, using polysorbate 80 and sorbitan monooleate 20 as surfactants. The NE presented an average size of 105.3 nm (±1.36) and a polydispersity index of 0.263 (± 0.004). The larvicidal bioassays against third-stage *Aedes aegypti* larvae produced an LC_50_ of 61.4 µg/mL at 48 hours and 26.8 µg/mL at 144 hours. The toxicity of NE in *Apis mellifera* was evaluated, proving that the formulated nanoemulsion did not present toxicity for this non-target species.

Viana et al. (2023) explored the larvicidal activity of a nanoemulsion formulated with an essential oil extracted from the leaves of *Xylopia ochrantha*, a plant native to the restinga ecosystem of Brazil [111]. The oil has a composition rich in sesquiterpenes, the main ones being germacrene D (17.8%), bicyclogermacrene (17.4%), and δ-elemene (13.9%). The extraction was performed by hydrodistillation, and the nanoemulsion was prepared using a low-energy method with sorbitan monooleate 80 and polysorbate 20. The NE presented an average droplet size of 74.5 nm (±1.939) and a polydispersity index of 0.271 (±0.007), remaining physicochemically stable for up to 180 days. Larvicidal bioassays against third-stage *Aedes aegypti* larvae produced an LC_50_ of 192.5 µg/mL at 48 hours. Furthermore, acute oral toxicity tests in *Danio rerio* (zebrafish), a non-target model organism, showed no adverse effects. Although the study did not compare the nanoemulsion to the corresponding free essential oil, it highlights the potential of this formulation as a safe and environmentally friendly bioinsecticide for the control of arbovirus vectors.

Subaharan et al. (2022) evaluated the larvicidal efficacy of nanoemulsions of *Trachyspermum ammi* essential oil and its main component, thymol, against *Ae. aegypti*. The plant, native to India, has seeds rich in thymol (54.22%), p-cymene (15.04%), and γ-terpinene (10.46%) [116–117]. The oil was extracted by hydrodistillation (1.8% yield), and the nanoemulsions were prepared with 5% of oil or thymol, 5% of Tween^®^ 80, and 90% of water by ultrasound at 50 °C. The nanoemulsions presented average sizes of 65 to 83 nm (oil) and 167 to 230 nm (thymol), with stability of up to 60 days. In bioassays with third-instar larvae, thymol nanoemulsion demonstrated greater efficacy (LC_50_ = 34.89 ppm), followed by oil (LC_50_ = 46.73 ppm), both outperforming conventional emulsions. Electron microscopy revealed damage to the larvae, and enzymatic analysis showed that thymol nanoemulsion inhibited 83.48% of acetylcholinesterase, while oil inhibited 53.62%, suggesting multiple mechanisms of action.

Gupta et al. (2022) investigated the larvicidal efficacy of a *Thymus vulgaris* essential oil nanoemulsion against *Ae. Aegypti* [118]. Thyme, a plant native to the Mediterranean, has antimicrobial and insecticidal properties, with main compounds such as 1,3,8-p-menthatriene (45.58%) and 2-ethyl-4,5-dimethyl-phenol (41.50%) [119]. The essential oil was extracted by steam distillation. The nanoemulsion was prepared by ultrasound, with a ratio of 1:0.5 (oil/surfactant, polysorbate 80) and Milli-Q water. It presented an average size of 52.18 nm (±4.53), a polydispersity index of 0.237 (±0.006), and thermal stability. In bioassays, third instar larvae exposed to concentrations between 5 and 150 ppm showed LC_50_ of 58.72 ppm after 24 hours. Morphological analysis revealed significant damage to the larvae, such as cuticular deformations, especially in the anal segment. The rapid release of the essential oil (91.68% in 48 hours) indicated high bioavailability, contributing to the efficacy of the nanoemulsion.

Bosly et al. (2022) developed an oil-in-water nanoemulsion containing *Santalum album* (sandalwood) essential oil and evaluated its larvicidal activity against *Ae. aegypti* [120]. The essential oil was extracted by hydrodistillation from the wood and roots of *Santalum album*, a tree native to India, East Asia, northern Australia, and the Hawaiian Islands [121]. This oil is rich in sesquiterpene alcohols, such as α-santalol (24.27%) and β-santalol (27.65%) [120,122]. The NE presented an average droplet size of 195.7 nm, a polydispersity index of 0.342, and a negative zeta potential (−20.1 mV). Third-instar larvae were exposed to concentrations ranging from 62.5 to 1500 ppm. The nanoemulsion showed LC_50_ values of 232.18 and 182.37 ppm after 24 and 48 hours, showing greater efficacy than that of the free essential oil, which required higher concentrations. Furthermore, the nanoemulsion significantly reduced the activity of the enzymes α-esterase, β-esterase, and glutathione S-transferase (GST) in the larvae, suggesting interference in the detoxification mechanisms and indicating the involvement of enzymatic pathways in the larvicidal action.

Almadiy and Nenaah (2022) developed an oil-in-water nanoemulsion containing *Origanum vulgare* essential oil, composed mainly of carvacrol, thymol, γ-terpinene, and p-cymene – monoterpenes widely present in species from the mediterranean and temperate regions of Asia [72,123]. The formulation was composed of EO, polysorbate 80 (surfactant), and purified water, and it was stabilized at 25 °C for 30 days. The nanoemulsion showed physicochemical stability with an initial average size of 64.1 nm (±6.3), polydispersity index of 0.21 (±0.04), and initial pH of 5.8. After 30 days, the average size increased to 71.8 ± 8.3 nm, with a polydispersity index of 0.18 ± 0.03 and pH of 4.9 ± 0.06. In larvicidal bioassays with *Ae. aegypti* (exposure: 24 hours), the nanoemulsion presented a LC_50_ of 13.9 µg/mL, higher than that of isolated EO (29.2 µg/mL) and pure terpenes (carvacrol: 31.9 µg/mL; thymol: 45.3 µg/mL; γ-terpinene: 50.4 µg/mL; *p*-cymene: 75.7 µg/mL). The 40 µg/mL dose of the nanoemulsion eliminated 100% of the larvae tested. No toxicity data for non-target organisms were reported.

Rodrigues et al. (2021) evaluated the larvicidal potential of nanoemulsions formulated with essential oils from two morphotypes of *Ayapana triplinervis*, a plant native to South America and widely distributed in Brazil, Ecuador, Peru, and other countries [124–125]. The plant material was collected in the state of Amapá (Brazil), and the morphotypes were differentiated by morphological characteristics and chemical composition determined by GC-MS. The morphotype A presented β-caryophyllene (45.93%) and thymohydroquinone dimethyl ether (32.93%) as the main constituents, while the morphotype B was dominated by the latter compound (84.53%). Nanoemulsions were obtained by the low-energy method, with average sizes of 101.4 nm (morphotype A) and 104.6 nm (morphotype B), polydispersity < 0.17, and zeta potentials between −19.3 and −27.7 mV. Third-instar larvae of *Aedes aegypti* were treated with concentrations of 50–150 µg/mL (A) and 20–100 µg/mL (B), with the morphotype B formulation being the most effective (LC_50_ = 35.57 µg/mL in 48 hours), surpassing the free essential oil. Acute oral toxicity tests in *Mus musculus* mice (2000 mg/kg) did not result in mortality, although inflammatory changes were observed in the lungs and liver.

Faustino et al. (2021) investigated the larvicidal potential of a nanoemulsion formulated with an essential oil extracted from the resin of *Protium heptaphyllum*, a species native to the Amazon region. The chemical composition of the oil was dominated by p-cymene (27.70%) and α-pinene (22.31%), compounds recognized for their insecticidal activity [126–127]. The nanoemulsion was obtained by a low-energy method, presenting ideal physicochemical characteristics, with an average diameter of 109.7 nm (±0.75), a polydispersity index of 0.29 (±0.007), and a zeta potential of −21.7 mV (±1.10), conferring stability to the system. The larvicidal tests were performed with third-stage larvae of *Ae. aegypti,* exposed to the samples for a period of 24 and 48 hours. The nanoemulsion demonstrated high efficacy, with a LC_50_ of 2.91 µg/mL after 48 hours, outperforming the free essential oil. Finally, tests with zebrafish revealed only mild changes in the gills at concentrations higher than those used in the bioassays, demonstrating low toxicity of the nanoemulsion.

Folly et al. (2021) developed a nanoemulsion with an essential oil from the leaves of *Annona acutiflora*, a plant native to tropical regions of South America, with occurrences recorded in Brazil, Colombia, Peru, Ecuador, and Venezuela. The chemical composition of the essential oil is rich in sesquiterpenes, with α-santalene, bicyclogermacrene, and α-zingiberene as the main constituents [128–129]. The nanoemulsion was prepared with essential oil, polysorbate 20, and distilled water using magnetic stirring and aqueous titration, followed by emulsification. The resulting formulation had an average size of 171.1 nm (±1.2), a polydispersity index of 0.171 (±0.011), and a zeta potential of −15.0 mV (±0.53). In larvicidal bioassays performed with third-instar larvae of *Ae. aegypti*, concentrations of 12.5, 25.0, and 50.0 ppm were evaluated, with exposure times of 24 and 48 hours. The LC_50_ values were 36 ppm (24 hours) and 21.2 ppm (48 hours), with maximum mortality of 100% being observed at the concentration of 50 ppm after 48 hours.

Martins et al. (2021) prepared a nanoemulsion containing essential oil from the leaves of *Aeollanthus suaveolens* to evaluate its larvicidal efficacy against *Ae. aegypti* and its toxicity to non-target organisms [130]. This species belongs to the Lamiaceae family, native to Africa, and is also distributed in the north and northeast of Brazil [131–132]. The main constituents of its essential oil are massoialactone (64.79%), linalool (7.83%), and (E)-β-farnesene (6.17%) [130]. The NE presented an average size of 104.3 nm (±0.47), a polydispersity index of 0.156 (±0.01), and a zeta potential of −13.63 mV (±0.83). The L3 larvae were treated with NE (100, 80, 60, 40, and 20 µg/mL) in a 100 mL container. An aqueous dispersion with the surfactant was used as the negative control and sbiotrin as the positive control. The larvicidal bioassay demonstrated that the concentration of 100 mg/mL resulted in 98% mortality in 24 hours and 100% after 48 hours. The LC_50_ was 54.23 mg/mL after 24 hours and 46.06 mg/mL at 48 hours.

De Oliveira et al. (2020) developed a nanoemulsion containing *Piper alatipetiolatum* essential oil and evaluated its ovicidal, larvicidal and pupicidal efficacy against *Aedes aegypti* [133]. This native brazilian species presented essential oil compositions mainly consisting of ishwarona (78.6%), ishwarol (8.2%), β-elemene (6.9%), selin-11-en-4α-ol (2.9%), and ishwaran (2.4%), which have been reported to possess biological activities such as larvicidal effects [134]. Based on this, a nanoemulsion was prepared and evaluated against eggs, L3 larvae, and pupae. The NE presented a size of 316 nm (±8), a polydispersity index of 0.44 (±0.01), and a zeta potential of −8.5 mV (±0.14). The essential oil showed lower ovicidal (19 to 100%), larvicidal (LC_50_ of 33.74 ppm), and pupicidal (LC_50_ of 65.06 ppm) activity when compared to those of the nanoemulsion containing this oil. The formulation showed higher ovicidal (47.7 to 100%), larvicidal (LC_50_ of 6.37 ppm), and pupicidal (LC_50_ of 9.33 ppm) activity against *Aedes aegypti.*

Rodriguez Amado et al. (2020) developed a nanoemulsion with 5% of essential oils extracted from the leaves of *Croton linearis*, a species widely distributed throughout the Americas [135–136]. The oil, with a yield of 1.5%, is mainly composed of eucalyptol (26.66%), sabinene (9.37%), and 10-epi-γ-eudesmol (6.83%) [135]. The NE presented an average size of 175 nm, a polydispersity index of 0.074, and a negative zeta potential (−27.02 mV). In bioassays, fourth-instar larvae of *Ae. aegypti* were exposed to concentrations of 10 to 100 µg/mL of the free essential oil and the nanoemulsion in distilled water. The nanoformulation presented an LC_50_ of 17.86 µg/mL after 24 hours, demonstrating superior efficacy to the free oil (LC_50_ = 64.24 µg/mL). Controls included surfactant-treated water and temephos (12 µg/L). Finally, toxicity was evaluated in Vero (*Chlorocebus sabaeus*) kidney cells and in adult Wistar rats via oral administration. The assays revealed no hemolytic, cytotoxic, or toxic effects, with IC_50_ above 2000 mg/kg, indicating a good environmental safety profile of the formulation.

Suresh et al. (2020) investigated the use of nanoemulsions containing *Crithmum maritimum* essential oil to control *Ae. aegypti*. The plant, typical of coastal areas, is widely distributed along the Mediterranean coast, including countries in Europe, North Africa, and regions of Western Asia [137–138]. Chemical analysis of the oil revealed compounds with insecticidal activity, such as dillapiole, myristicin, γ-terpinene, and thymol methyl ether. The nanoemulsion was prepared using a low-energy method, combining 5% of essential oil, 5% of polysorbate 20, and 90% of water. The most stable formulation was obtained with a 1:3 ratio between oil and surfactant, presenting droplets with a diameter between 50 and 70 nm and a zeta potential of −18.3 mV, indicating good stability. In biological tests, the nanoemulsion demonstrated efficacy against the immature stages of the vector. The LC_50_ values ranged from 27.467 to 63.134 µL/L for larvae and from 49.662 to 96.436 µL/L for pupae, with greater toxicity observed in the larval stage. In addition, tests were conducted with *Artemia salina*, a non-target species, which showed mortality below 10%, even at high concentrations of the formulation. This suggests a low ecotoxicological risk and potential for safe application in mosquito control.

Ferreira et al. (2020) investigated the larvicidal effects of a nanoemulsion containing essential oil from *Siparuna guianensis,* a native Brazilian plant rich in oxygenated sesquiterpenes, the major components being curzerenone (18.86%) and α-muurolol (11.75%), in addition to the presence of the compounds curzerene and γ-muurolene [112]. The oil was extracted by hydrodistillation and incorporated into the nanoemulsion using a low-energy method, without heating and without solvents, using polysorbate 80 as the surfactant. The nanoemulsion had an average droplet size of 176.0 nm (±12.3) and a polydispersity index of 0.38 (±0.004). In the bioassays, third-stage larvae of *Aedes aegypti* were exposed for 24 and 48 hours. The nanoemulsion demonstrated significantly superior efficacy to the free essential oil, with CL_50_ values of 24.75 µg/mL after 24 hours and CL_90_ of 54.17 µg/mL after 48 hours, while the free essential oil presented CL_50_ of 86.52 µg/mL and CL_90_ of 134.81 µg/mL after 24 hours, and CL_50_ of 82.81 µg/mL and CL_90_ of 128.39 µg/mL after 48 hours. It is noteworthy that the formulation was not tested in non-target organisms.

Kaur et al. (2019) developed an optimized nanoemulsion containing *Eucalyptus globulus* essential oil for larvicidal control of *Ae. Aegypti* [139]. The plant, originally from Australia, is widely cultivated in tropical and subtropical regions. Although the oil used was not characterized in the study, its composition is known to be rich in compounds such as eucalyptol (1,8-cineole), gamma-terpinene, globulol, and gamma-pinene, with eucalyptol being considered the main agent responsible for the larvicidal action [139–140]. The nanoemulsion formulation was obtained in a 1:2 (v/v) ratio between the oil phase (oil + polysorbate 20) and water, prepared by magnetic stirring followed by ultrasonication (40 kHz). This formulation demonstrated physicochemical stability, with good optical transparency and absence of phase separation. Characterization by TEM revealed spherical droplets with sizes between 20 and 40 nm. In the larvicidal bioassay, fourth-instar larvae of *Ae. aegypti* were exposed to different concentrations of the nanoemulsion. The dose of 70 µg/mL was the most effective, promoting 100% mortality within 24 hours. The LC_50_ and LC_90_ values were 60.33 and 92.29 µg/mL, respectively.

Mishra et al. (2018) developed a nanoemulsion using *Azadirachta indica* (neem) oil, urea, and Tween^®^ 20 as surfactant, by microfluidization [141]. Neem, originating from the Indian subcontinent and widely distributed in tropical and subtropical regions, has as its major constituents azadirachtin, nimbin, and salanin, recognized for their bioinsecticidal properties. The nanoemulsion presented an average droplet size of 12.3 ± 0.06 nm, polydispersity index of 0.249, and zeta potential of −21.7 ± 1.22 mV. In larvicidal bioassays with L3 larvae of *Aedes aegypti* (exposure: 24 hours), the LC_50_ value was 99.26 µg/mL, lower than that of the neem oil alone (123.59 µg/mL) and Tween 20 (169.30 µg/mL), indicating greater efficacy of the nanoformulation. No toxicological tests on non-target organisms were reported.

Balasubramani et al. (2017) developed a nanoemulsion with essential oil from *Vitex negundo* L., an Asian plant used for medicinal purposes [69,142]. The oil, rich in compounds such as 2*R*-acetoxymethyl-1,3,3-trimethylcyclohexanol (27.2%), nerolidol (14.6%), and β-caryophyllene (11.9%), was extracted by hydrodistillation (0.5% yield) [69,143]. The nanoemulsion was formulated with 5% essential oil, 5% polysorbate 80, and 90% water, resulting in droplets smaller than 200 nm and stability for 30 days. In bioassays with *Ae. aegypti* larvae, concentrations of 25 to 400 ppm of the nanoemulsion and free oil showed greater larvicidal efficacy of the nanoemulsion, with LC_50_ of 28.84 µg/mL (second instar, 24 hours) and 43.29 µg/mL (third instar, 24 hours), compared to the free oil, which presented LC_50_ of 77.35 µg/mL and 56.13 µg/mL, respectively.

Botas et al. (2017) developed a nanoemulsion based on the essential oil of *Baccharis reticularia*, a plant native to Brazil, also found in Paraguay, Bolivia, and Argentina. The oil is mainly composed of mono- and sesquiterpenes, including ᴅ-limonene, a key precursor in the biosynthesis of monoterpenes [73,144]. The formulation was obtained by a low-energy method, presenting an average droplet diameter of 92.9 nm (±0.4), polydispersity index of 0.412 (±0.009), and zeta potential of −20.4 mV (±0.6). In larvicidal tests with fourth-instar larvae of *Ae. aegypti* for 24 hours and 48 hours, the LC_50_ was 221.273 μg/mL and 144.685 μg/mL, respectively. Histological damage, such as changes in intestinal cells, was observed, indicating a potentially harmful effect on the larvae. Tests in non-target species were not performed.

Oliveira et al. (2016) prepared a nanoemulsion based on *Pterodon emarginatu*s essential oil and evaluated its larvicidal activity [145]. This species *Pterodon emarginatus*, popularly known as white sucupira or faveira, is a tree which is native to the Brazilian Cerrado [145–146]. The diterpenes methyl 6α,7β-dihydroxyvouacapan-17-β-oate (MHV), geranylgeraniol, and the sesquiterpene β-caryophyllene, reported in the literature for their larvicidal activity, stand out as constituents [145]. The formulation was tested against fourth-instar *Ae. aegypti* larvae at concentrations ranging from 12.5 to 250 µg/mL, with assessments conducted at 24 and 48 hours. Maximum mortality occurred at 250 µg/mL, while a notable increase in efficacy at 75 ppm was observed over extended exposure. Toxicological evaluation in *Mus musculus* revealed no signs of systemic toxicity, supporting the selective action of the formulation and safety for non-target organisms.

The studies (Table 1) suggest that nanoemulsions enhance the larvicidal properties of essential oils against *Ae. aegypti*, with efficacy varying according to the plant, composition, and formulation method. Many nanoemulsions have demonstrated stability over time and low toxicity to non-target species, making them a promising and sustainable solution for mosquito control.

**Table 1:** Overview of studies on the use of nanoemulsions with essential oils in the larvicidal control of *Aedes aegypti*.

Species	Nanoemulsion characterizations (size, polydispersity index, and zeta potential)	Larval stage of the *Ae. aegypti* tested	Time (hours)	LC_50_	LC_90_	Ref.

*Murraya koenigii*	size = 140.00 nm PdI = 0.24 ZP = −16.10 mV	L3	24	11.80 µg/mL (lab)	22.60 µg/mL (lab)	[108]
				12.50 µg/mL (field)	21.60 µg/mL (field)	
*Ocimum basilicum*	size = 244.60 to 280.40 PdI = Less than 0.25 ZP = −15.70 to −18.60 mV	L4	24	42.15 µg/mL	50.35 µg/mL	[76]
			48	40.94 µg/mL	48.87 µg/mL	
*Ocotea indecora*	size = 105.30 nm (±1.36) PdI = 0.26 (±0.004) ZP = −23.8 mV (±2.01)	L3	48	61.40 µg/mL	ND	[113]
			144	26.80 µg/mL		
*Xylopia ochrantha*	size = 74.50 nm (±1.939) PdI = 0.27 (±0.007) ZP = −25.15 mV (±0.65)	L3	48	192.50 µg/mL	ND	[111]
*Trachyspermum ammi*	size = 65.00 nm (±0.7) to 83.00 nm (±0.09) PdI = between 0.18 (±0.003) and 0.20 (±0.07)	L3	24	46.73 µg/mL	ND	[117]
*Thymus vulgaris*	size = 52.18 nm (±4.53) PdI = 0.23 (±0.006) ZP = 1.62 mV (±0.052)	L3	24	58.70 µg/mL	ND	[118]
*Santalum album*	size = 195.70 nm PdI = 0.34 ZP = −20.10 mV	L3	24	232.18 µg/mL	ND	[120]
			48	182.37 µg/mL		
*Origanum vulgare*	size = 64.10 nm (±6.3) PdI = 0.21 (±0.04)	L3	24	13.90 µg/mL	21.60 μg/mL	[72]
							*Ayapana triplinervis*	size = 88.83 nm (±0.948) to morphotype A and 99.637 nm (±0.529) to morphotype B PdI = 0.138 (±0.012) to morphotype A and 0.213 (±0.011) to morphotype B ZP = −23.20 mV (±0.458) to morphotype A and −22.00 mV (±1.153) to morphotype B	L3	24	96.23 µg/mL (morphotype A) and 44.76 µg/mL (morphotype B)	202.04 µg/mL (morphotype A) and 100.47 µg/mL (morphotype B)	[125]
48	87.43 µg/mL (morphotype A) and 35.57 µg/mL (morphotype B)	177.21 µg/mL (morphotype A) and 117.45 µg/mL (morphotype B)											
*Protium heptaphyllum*	size = 109.7 nm (±0.75) PdI = 0.29 (±0.007) ZP = −21.7 mV (±1.10)	L3	24	2.91 µg/mL	0.17 µg/mL and 8.87 µg/mL	[127]
			48	12.44 µg/mL		
*Annona acutiflora*	size = 171.10 nm (±1.2) PdI = 0.17 (±0.011) ZP = −15.0 mV (±0.53)	L3	24	36.00 µg/mL	ND	[128]
			48	21.20 µg/mL		
*Aeollanthus suaveolens*	size = 104.83 nm (±0.47) PdI = 0.16 (±0.01) ZP = −13.63 mV (±0.83)	L3	24	54.23 µg/mL	96.96 µg/mL	[130]
			48	46.06 µg/mL	75.31 µg/mL	
*Piper alatipetiolatum*	size = 316.0 nm (±8) PdI = 0.44 (±0.01) ZP = −8.50 mV (±0.14)	L3	24	6.370 µg/mL	21.70 µg/mL	[133]
*Croton linearis*	size = 175.0 nm PdI = 0.074 ZP = −27.02 mV	L4	24	17.86 μg/mL	ND	[135]
*Crithmum maritimum*	size = 50.0 and 70.0 nm ZP = −18.3 mV	Larval stage	24	27.47 to 48.94 µL/L	65.80 to 105.14 µL/L	[138]
		Pupae		63.13 µL/L	121.95 µL/L	
*Siparuna guianensis*	size = 176.0 nm (±12.3) PdI = 0.381 (±0.004)	L3	24	24.75 μg/mL	75.24 μg/mL	[112]
			48	NI	54.17 μg/mL	
*Eucalyptus globulus*	sizes between 20 and 40 nm	L4	12	60.33 µg/mL	92.29 µg/mL	[139]
*Azadirachta indica*	size = 12.30 nm (±0.06) PdI = 0.25 ZP = −21.70 mV (±1.22)	L3	24	99.26 µg/mL	ND	[141]
*Vitex negundo*	size = smaller than 200 nm	L2	24	28.84 µg/mL	ND	[69]
		L3		43.29 µg/mL		
*Baccharis reticularia*	size = 92.9 nm (±0.4) PdI = 0.412 (±0.009) ZP = −20.4 mV (±0.6)	L4	24	221.27 μg/mL	ND	[73]
			48	144.69 μg/mL		
*Pterodon emarginatus*	size = 135.8 nm (±0.2) PdI = 0.173 (±0.002) ZP = −27.2 (±0.6)	L4	24	–	ND	[145]
			48	34.75 μg/mL		

ND: not defined; PdI: polydispersity index; ZP: zeta potential.

#### Repellent properties

**Figure 4:**
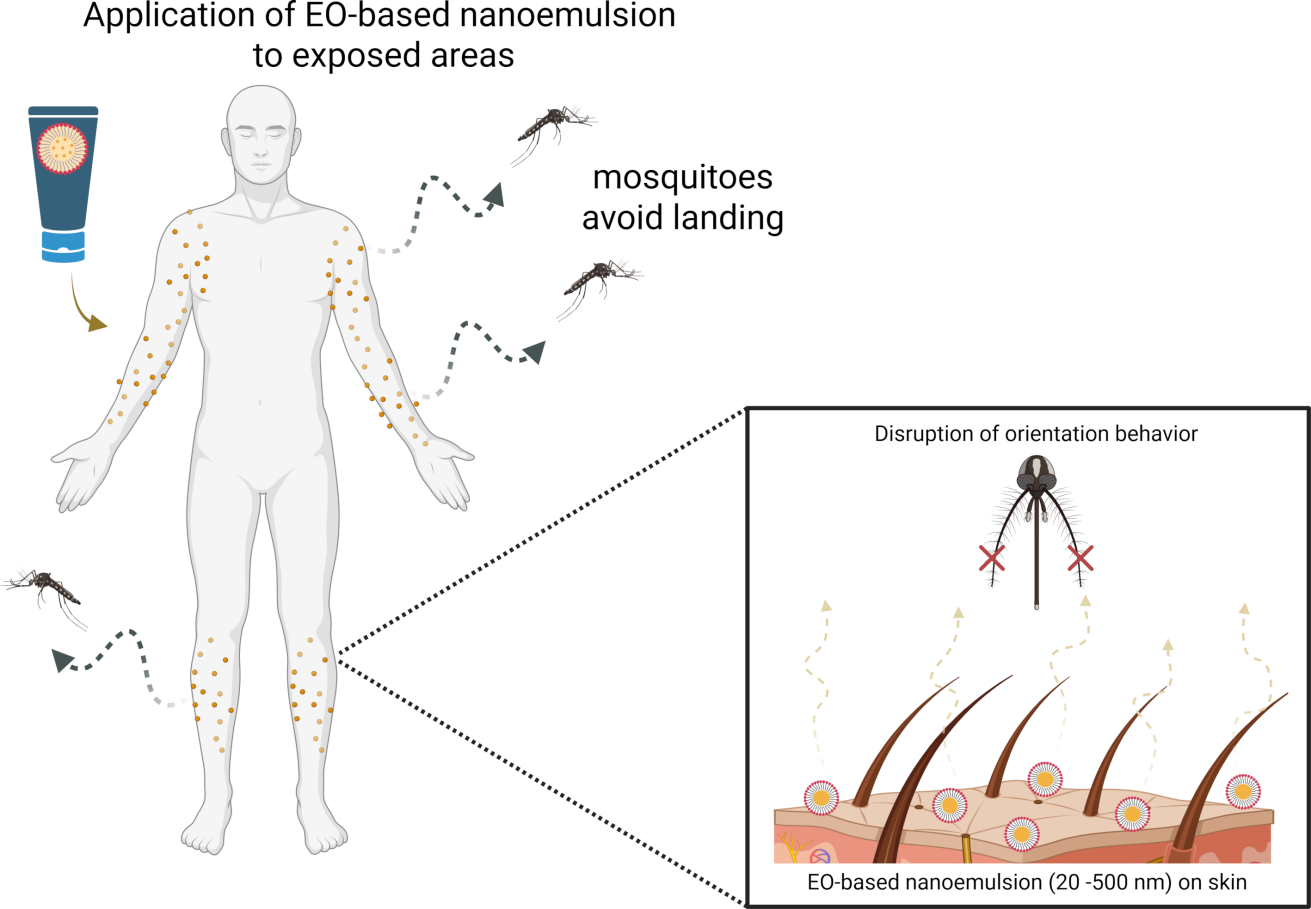
Schematic representation of the repellent mechanism of essential oil-based nanoemulsions against *Aedes aegypti* mosquitoes. Created in BioRender. Rocha Formiga, F. (2025) https://BioRender.com/7d1odbe. This content is not subject to CC BY 4.0.

Essential oils have repellent properties against mosquitoes such as *Ae. aegypti.* Their efficacy has been enhanced when incorporated into nanoemulsions [147–150]. These nanostructured systems improve the stability, volatility, and skin permeation of the active compounds, prolonging the repellent effect due to the controlled release of these compounds [151–152]. The mechanism of action may involve interference with the olfactory system of the mosquitoes, masking host signals and disrupting orientation behavior [147] (Figure 4).

Despite the increased efficacy and potential of nanoemulsions containing essential oils against this vector, only three studies have evaluated the repellent efficacy of these nanoformulations in the last 10 years, the data for which are compiled in Table 2 and detailed below.

**Table 2:** Recent studies (last 10 years) evaluating the repellent efficacy of nanoemulsions containing essential oils against *Aedes aegypti*.

Species	Nanoemulsion characterizations (size, polydispersity index, and zeta potential)	Concentration	Protection (percentage)	Time	Release	Ref.

*Protium heptaphyllum*	size = 109.00 nm (±0.75) PdI = 0.29 (±0.007) ZP = −21.70 mV (±1.10)	200 µg/mL	77.67%	3 h	ND	[150]
*Myristica fragrans*	size = 217.00 nm PdI = 0.25 ZP = −44.20 mV	ND	87.81%	8 h	53.3% in 8 h and 71.5% after 24 h	[148]
*Mentha piperita* and *Eucalyptus globulus*	size = 137.00 nm PdI = 0.28 ZP = −43.10 mV	ND	85.40%	8 h	51.7% after 8 h and 69.6% after 24 h	[149]

ND: not defined; PdI: polydispersity index; ZP: zeta potential.

In addition to the larvicidal efficacy reported by Faustino and collaborators (2021), the authors also evaluated the efficacy of a nanoemulsion containing *Protium heptaphyllum e*ssential oil [150]. This demonstrated significant repellent activity against adult females of *Ae. aegypti*. The repellency tests were conducted in a laboratory under controlled conditions, where samples of the nanoemulsion were applied to simulated human skin (using an artificial membrane) and exposed to previously fasted adult females of *Ae. aegypti*. The formulation, at a concentration of 200 µg/mL, provided a protection rate of 77.67% over 180 minutes (3 hours), demonstrating prolonged effect in relation to the free essential oil, which showed rapid loss of activity due to volatilization.

Mohd Narawi et al. (2020) developed a nanoemulsion with nutmeg essential oil (*Myristica fragrans*), an aromatic plant native to the Moluccan Islands and widely cultivated in tropical regions [148,153]. The oil, rich in α-pinene, safrole, and terpinen-4-ol, was encapsulated with Montanov 82, glycerol, and distilled water, resulting in droplets of approximately 217 nm, with a PdI of 0.248 and a zeta potential of −44.2 mV. The incorporation efficiency reached 85.4%, according to gas chromatography analysis. In the tests, Sprague Dawley rats received the formulation applied to their backs before being exposed to 50 female *Ae. aegypti* for 8 hours. The average protection was 87.81%, surpassing the pure oil (54.57%) and falling below DEET (100%). The study also demonstrated controlled release of the active ingredient, with 53.3% released in 8 hours (33.31 mg/mL) and 71.5% after 24 hours. Finally, cytotoxicity tests were conducted, wherein the nanoemulsion maintained high cellular compatibility, with viability greater than 97% in murine fibroblasts (L929) up to concentrations of 400 µg/mL.

Finally, Mohammadi and colleagues (2020) evaluated the repellent efficacy of a nanoemulsion containing *Mentha piperita* and *Eucalyptus globulus* essential oils against *Ae. aegypti* [149]. These oils are widely found in tropical and temperate regions and are used for their aromatic and medicinal properties [154]. The chemical profiles of the oils include major constituents such as ᴅ-limonene, thymol, and carvacrol in *M. piperita*, and 1,8-cineole and γ-terpinene in *E. globulus* [149]. Thus, the oils were combined and incorporated using the high-pressure homogenization method, resulting in droplets with an average size of 137 nm, PdI of 0.279, and zeta potential of −43.1 mV.

Unlike previous authors, they verified the repellent efficacy in humans. The nanoemulsion was applied to the skin of volunteers before exposure to 50 female *Ae. aegypti* for 8 hours. The treatment obtained 85.4% protection, compared to that of the pure essential oil, which had a 57.4% efficacy. In addition, the formulation demonstrated a controlled release of the active compounds, with 51.7% released after 8 hours and 69.6% after 24 hours. Regarding safety, the cell viability of murine fibroblast cultures (L929 cells) was greater than 90% up to concentrations of 400 µg/mL, indicating low toxicity to human cells.

In summary, nanoemulsions improved the repellent efficacy of essential oils against *Aedes aegypti*, with efficacy varying according to the plant and the composition of the essential oils. Furthermore, these nanoemulsions demonstrate low toxicity given the high cell viability in tests with murine fibroblasts and safety in rats and humans, which makes them a promising and safe option for mosquito control.

### General discussion and final considerations

This review gathered recent advances in the use of nanoemulsions containing essential oils as an alternative and eco-friendly tool for controlling *Aedes aegypti*.

#### Botanical diversity and chemical composition of essential oils

Regarding the plant species used, a wide botanical and geographic diversity was observed, including plants native to South America, Asia, and Europe. This variety is reflected in the chemical richness of the essential oils used, in which compounds such as thymol, carvacrol, β-caryophyllene, citronellol, and α-santalol stand out as the main agents responsible for the larvicidal and repellent effects. These metabolites act in a multifactorial manner, including inhibition of acetylcholinesterase, generation of reactive oxygen species, morphological damage, and impairment of the larval cuticular barriers.

In addition to the technical and methodological gaps, the challenges associated with the seasonality of the plant species used stand out. The variation in the chemical composition of essential oils throughout the year, influenced by environmental and phenological factors, can significantly impact the biological efficacy of formulations and hinder standardization on an industrial scale. This factor represents a significant practical obstacle, especially when seeking reproducibility of results and stability of products over time.

#### Surfactants composition of nanoemulsions

Among the technical and formulation aspects observed, the recurring use of nonionic surfactants, such as polysorbate 20, polysorbate 80, and sorbitan monoleate 80, stands out. These agents are widely used due to their low toxicity and high emulsifying capacity [155]. These agents promote the emulsification and stabilization of nanoemulsions. They can also influence aspects such as entrapment efficiency and release of active ingredients, aspects that still lack standardization in the studies evaluated. Additionally, nonionic surfactants can present zeta potential values below −30 mV, conferring steric stability on part of their chains, as observed in most studies reported in dilutions in purified water. Values above −30 mV may reflect EO compounds with ionizable groups, which confer electrical double layers and/or a diluent rich in salts, not reported by the authors [155]. Furthermore, although not evaluated by studies, the concentration and size of surfactant chains may influence droplet size distribution [156].

#### Challenges in the development of repellent nanoformulations

Despite the emphasis on the larvicidal activity, only three studies have addressed the repellent potential of these formulations, revealing a critical gap in the literature. The scarcity of data on repellency may be linked to the limitations imposed by ethics committees when conducting tests with humans and animals. In view of this, there is an urgent need to develop alternative and ethical approaches, such as predictive computational models (QSAR), diffusion systems with synthetic membranes, and automated bioassays to assess mosquito behavior.

The lack of studies on skin permeation and photostability also represents an important limitation. Repellent products intended for topical application require validation regarding their ability to cross the epidermal barrier and resist degradation induced by sunlight. These characteristics are essential to ensure prolonged efficacy and user safety, especially under real-world conditions of use. The lack of such tests in the publications analyzed reveals a critical point that needs to be overcome in order to enable the translation of these nanostructured systems for commercial applications.

Another fundamental point is the lack of data on incorporation efficiency and in vitro release tests. These variables are crucial to ensuring the stability of the system, the maintenance of the content of bioactive compounds over time, and the correlation between the release of the active ingredient and the time of protection or larvicidal action. The lack of methodological standardization in this regard compromises not only the reproducibility of studies, but also the scalability of the products developed.

#### Assessment in non-target species

Additionally, the environmental and biological safety of formulations is a dimension that deserves greater attention. Although some authors have evaluated toxicity in non-target organisms such as *Danio rerio*, *Apis mellifera*, *Mus musculus*, *Galleria mellonella*, and *Artemia salina*, these tests are still isolated and do not predominate in the literature. Similarly, some studies have performed in vitro tests with human and murine cell lines, such as HaCaT (human keratinocytes) and L929 (murine fibroblasts), demonstrating low cytotoxicity at certain concentrations. However, these cellular safety assessments are still not prevalent among the reviewed studies, being treated more as complements than as fundamental validation steps.

Finally, even in view of the demonstrated potential, nanotechnology products such as nanoemulsions face considerable translational barriers. The lack of clear and specific regulations for the registration of nanostructured formulations for entomological purposes makes their regulation and commercialization difficult. This regulatory gap generates legal uncertainty and limits the advancement of innovative technologies in the market. Therefore, it is essential that regulatory frameworks be updated and adapted to contemplate the specific safety, efficacy and quality requirements of these new technological platforms.

Therefore, although nanoemulsions with essential oils show promising performance in controlling *Aedes aegypti*, the consolidation of this strategy requires an interdisciplinary effort aimed at standardizing critical parameters, deepening the mechanisms of action, validating safety and efficacy, and overcoming technical, regulatory, and environmental barriers. Future studies should prioritize the integration of physicochemical, biological, and toxicological data, as well as strategies for standardizing plant raw materials to ensure the development of effective, safe, and sustainable products.

## Conclusion

The use of nanoemulsions based on essential oils is emerging as a viable and environmentally responsible alternative for controlling the *Aedes aegypti* vector. In addition to gathering evidence on their effectiveness, this review highlights the need for a paradigm shift in the way nanobiotechnological products are developed and evaluated. The future of this approach will depend not only on technical and scientific advances, but also on a regulatory environment prepared to welcome innovations with safety, transparency, and real applicability. This will consolidate the foundations for the development of safer and more efficient solutions that are compatible with current global public health challenges.

## Data Availability

Data sharing is not applicable as no new data was generated or analyzed in this study.
